# Lagos Bat Virus Infection Dynamics in Free-Ranging Straw-Colored Fruit Bats (*Eidolon helvum*)

**DOI:** 10.3390/tropicalmed2030025

**Published:** 2017-07-08

**Authors:** Richard D. Suu-Ire, Anthony R. Fooks, Ashley C. Banyard, David Selden, Kofi Amponsah-Mensah, Silke Riesle, Meyir Y. Ziekah, Yaa Ntiamoa-Baidu, James L. N. Wood, Andrew A. Cunningham

**Affiliations:** 1 Department of Animal Biology and Conservation Science, University of Ghana, P.O. Box LG 571, Legon, Accra, Ghana; mak2kofi@gmail.com (K.A.-M.); sasr3@cam.ac.uk (S.R.); ynbaidu@ug.edu.gh (Y.N.-B.); 2 Veterinary Services Department, Ministry of Food and Agriculture, P. O. Box M 161, Accra, Ghana; meyir73@gmail.com; 3 Wildlife Division of the Forestry Commission, P.O. Box M239, Accra, Ghana; 4 Wildlife Zoonoses and Vector-Borne Diseases Research Group, Animal and Plant Health Agency (APHA), Weybridge, Woodham Lane, New Haw, Addlestone, Surrey KT15 3NB, UK; tony.fooks@apha.gsi.gov.uk (A.R.F.); Ashley.Banyard@apha.gsi.gov.uk (A.C.B.); David.Selden@apha.gsi.gov.uk (D.S.); 5 Department of Clinical Infection, Microbiology & Immunology, Institute of Infection and Global Health, University of Liverpool, Liverpool LP69 7ZX, UK; 6 Cambridge Infectious Diseases Consortium, Department of Veterinary Medicine, University of Cambridge, Madingley Road Cambridge CB3 0ES, Cambridge, UK; jlnw2@cam.ac.uk; 7 Institute of Zoology, Zoological Society of London, Regent’s Park, London NW1 4RY, UK

**Keywords:** bat, rabies, *Eidolon helvum*, Lagos bat virus, seroprevalence, lyssavirus, Ghana

## Abstract

Bats are key species for ecological function, but they are also reservoirs of zoonotic agents, such as lyssaviruses that cause rabies. Little is known about the maintenance and transmission of lyssaviruses in bats, although the observation of clinically sick bats, both in experimental studies and wild bats, has at least demonstrated that lyssaviruses are capable of causing clinical disease in bat species. Despite this, extensive surveillance for diseased bats has not yielded lyssaviruses, whilst serological surveys demonstrate that bats must be exposed to lyssavirus without developing clinical disease. We hypothesize that there is endemic circulation of Lagos bat virus (LBV) in the straw-coloured fruit bat (*Eidolon helvum*) in Ghana, West Africa. To investigate this further, longitudinal blood sampling was undertaken quarterly between 2012 and 2014 on wild *E. helvum* at two sites in Ghana. Serum samples were collected and tested for LBV-neutralizing antibodies using a modified flourescent antibody virus neutralisation (FAVN) assay (*n* = 294) and brains from moribund or dead bats were tested for antigen and viral RNA (*n* = 55). Overall, 44.7% of the 304 bats sampled had LBV-neutralising antibodies. None of the brain samples from bats contained lyssavirus antigen or RNA. Together with the results of an earlier serological study, our findings demonstrate that LBV is endemic and circulates within *E. helvum* in Ghana even though the detection of viral infection in dead bats was unsuccessful. Confirmation that LBV infection is endemic in *E. helvum* in Ghana is an important finding and indicates that the potential public health threats from LBV warrant further investigation.

## 1. Introduction

Wildlife populations constitute a large and largely undefined reservoir of infectious agents, some of which are zoonotic [1,2,3]. Infectious pathogens that originate in wild animals have become increasingly important globally, as they have had substantial impacts on human health, agricultural production, wildlife-based economies and wildlife conservation. Bats (*Chiroptera*) represent a large group of wildlife, comprising ~20% of the 4600 mammalian species recognized to date [4]. Bats have several key ecological functions, including pollination, seed dispersal and insect control [5]. However, bats have also been implicated as the reservoir hosts for numerous emerging infectious diseases. Increasingly, bats have been recognized as important reservoir hosts for viruses that have the potential to cross species barriers to infect humans, domestic and wild mammals [4,6].

Lyssaviruses (family *Rhabdoviridae*, genus *Lyssavirus*) are the aetiological agents that cause the disease rabies—an acute, progressive, viral encephalitis that is almost invariably fatal [7]. Most lyssaviruses are carried by bats, but the association of these viruses with bats is complex and poorly understood [8]. Rabies virus (RABV) is found in terrestrial carnivores globally, but it is only found in bats in the Americas, where it is endemic in frugivorous, insectivorous and hematophagous bats [8]. In contrast, the other 13 known lyssavirus species have only been detected in the Old World, predominantly in bat species although infection of terrestrial carnivores has been documented for some lyssavirus species [9]. Globally, the most significant public health and veterinary burden caused by lyssaviruses is dog-mediated transmission of RABV, but instances of other lyssavirus infection of humans or domesticated animals have been reported on rare occasions following spill-over events [8,10].

One bat lyssavirus that has been associated with the infection of terrestrial carnivores is Lagos bat virus (LBV), a virus most commonly associated with infection of African fruit bats. Fruit bats of several species have been identified as reservoir hosts for LBV, with spill-over infections reported in dogs, cats and a mongoose [11,12,13,14]. Notably, LBV has never been reported in human cases of rabies or any other case of human meningoencephalitis. Diagnostic tests that can differentiate between lyssaviruses are not available in the majority of rabies endemic areas, therefore clarity surrounding the impact of non-RABV lyssaviruses, such as LBV, is lacking. High seroprevalence against lyssaviruses has been reported in apparently-healthy bats, including for LBV in the straw-colored fruit bat (*E. helvum*) in Ghana [15]. While the ecology, pathogenesis and pathology of RABV in terrestrial animals and humans is partially understood [7], knowledge on the other lyssavirus species is scant. In this study, investigations into the presence of LBV infection in a natural host, *E. helvum*, were made through post-mortem sampling of moribund and dead bats as well as through serological assessment of samples for LBV neutralizing antibodies.

## 2. Material and Methods

### 2.1. Ethics Statement

The study was approved by the Institutional Review Board of the Nuguchi Memorial Institute for Medical Research of the University of Ghana (NMIMR-ITB CPN 002/13-14 IORG0000908) and the Ethics Committee of the Zoological Society of London (WLE/0638).

### 2.2. Study Sites

**Figure 1:**
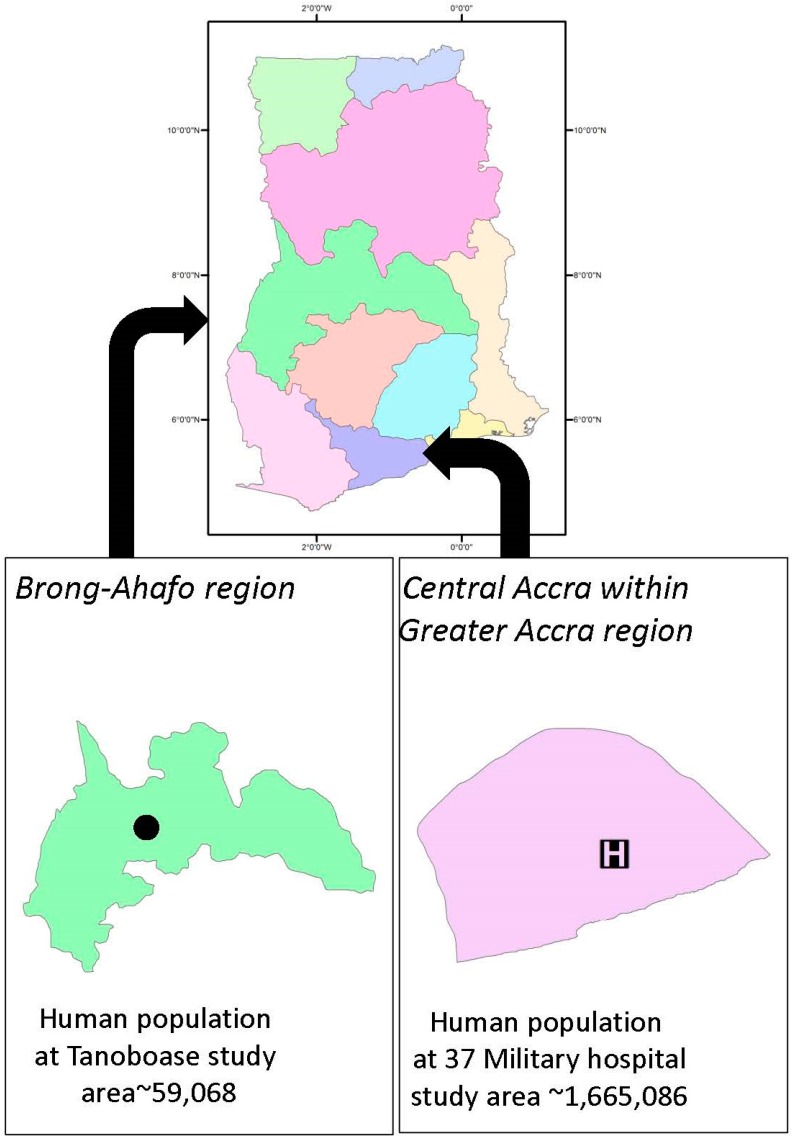
Locations at which wild straw-colored fruit bats (*Eidolon helvum*) were captured and sampled.

This study was undertaken at two *E. helvum* roost sites in Ghana, West Africa. The roosts comprised between approximately 3000 to 1,000,000 bats each, depending on year and season. One roost was located in Greater Accra (05°35.192′ N, 000°11.053′ W), within the grounds of a large general infirmary (37-Military Hospital) in the center of the city. The other roost was in Tanoboase in the Brong-Ahafo region (07°38′ N, 01°11.54′ W), within a protected forest area (Figure 1). The site in Tanoboase, known locally as Tano Sacred Grove, was developed for ecotourism in 1996 and is visited by both local and international tourists. The site also hosts an annual Apoo festival (a time of spiritual cleansing held in April–May).

### 2.3. Study Design

#### Sera Collection for Lagos Bat Virus Antibody Prevalence in Wild *Eidolon helvum*

Wild bats were captured using mist nets from 2012 to 2014 inclusive, as detailed in Table 1. Sampling in June/July was aimed to correspond with late-stage pregnancy. In July, the majority of the bats in each roost had migrated to alternative, as yet unknown, sites. By November, most bats had returned to each roost. Captured bats were housed individually in cloth bags for short time periods before being processed. The sex and sexual maturity of each bat were assessed based on body size (body mass and forearm length were measured routinely) and observation of external genitalia and mammary tissue. Approximately 1 mL of blood was collected in micro-tubes (SARSTEDT, Germany) from the propatagial vein of each individual, as described previously [15]. Blood samples were centrifuged at 6000× *g* for 15 min and the separated sera were stored at −70 °C until required. Each bat was micro-chipped (using the Trovan animal identification system, Electronic Identification Systems, Germany) to enable identification in case of recapture. Bats were released at the site of capture immediately following sampling, after ensuring haemostasis at the site of venipuncture.

### 2.4. Collection and Examination of Dead Bats

Members of the public who frequented the areas under the roosts were asked to report any dead or sick bats they found. Also, during each sampling visit, searches for dead or sick bats were undertaken in the immediate vicinity of the roosts. Sick bats were euthanised using intramuscular ketamine and medetomidine, followed by exsanguination via cardiac puncture. Bat carcasses were stored in a freezer at −70 °C until necropsy. At necropsy, a range of tissues, including brain, was collected and stored fixed or frozen for later investigation.

### 2.5. Laboratory Investigations

#### 2.5.1. Antigen Detection Using the Fluorescent Antibody Test (FAT)

Fluorescein isothiocyanate (FITC)-conjugated anti-rabies nucleocapsid polyclonal antibody conjugate (Fujirebio Diagnostics, Malvern, PA, USA) was used diluted 1:40 in 0.1 M phosphate buffered saline( PBS) pH 7.2 to stain virus antigen in acetone-fixed impression smears taken from the hippocampus, cerebellum and brain stem of bats found dead or sick (following euthanasia), as described previously [16]. Slides were examined using a fluorescence UV microscope. LBV-infected mouse brain material was used as a positive control for comparison of antigen distribution.

#### 2.5.2. Detection of LBV-Neutralizing Antibodies (mFAVN)

The presence of antibodies capable of neutralizing LBV in collected bat sera was assessed using a modified version of the fluorescent antibody virus neutralization assay (mFAVN), as described previously [2,15]. Briefly, lineage B LBV [17] was prepared to a working concentration of 100 tissue culture infective doses (TCID)_50_/50 μL. Both negative sera from an unvaccinated dog and positive anti-LBVNig56 rabbit serum samples were used as serological controls for the assay. Each test serum sample was diluted three-fold (starting with a 1:9 dilution) in minimum essential medium (MEM), and incubated with the viral inoculum for 1 h at 37 °C before being added to BHK-21 cells in suspension in a 96-well plate. Following incubation for 48 h at 37 °C, the plates were fixed in 80% chilled acetone for 20 min, and stained with FITC-labelled anti-rabies polyclonal antibody (Fujirebio Diagnostics, Malvern, PA, USA). Fluorescent foci present in cells infected with LBV were visualized at 488 nm using a fluorescence UV microscope. The lack of accurately-titred control sera meant that all neutralizing antibody levels were expressed as D_50_ values. The D_50_ for each sample is the dilution endpoint at which 50% of the wells showed the presence of virus. D_50_ values were calculated using the Spearman-Karber method. A serological cut-off was assigned according to neutralization of control sera and a back titration of input virus. 

#### 2.5.3. Detection of LBV RNA Using Molecular Methods

Total RNA was extracted from brain tissue samples using Trizol (Invitrogen) following the manufacturer’s protocol. A pan-lyssavirus SYBR Green real-time (q) RT-PCR [18] and a pan-lyssavirus conventional hemi-nested-RT-PCR [19] were used to determine the presence or absence of viral RNA.

## 3. Statistical Analysis

The chi-squared test was used to investigate if there were significant differences in seroprevalence according to the age, sex and roost location of bats.

## 4. Results

### 4.1. LBV Seroprevalence

**Figure 2:**
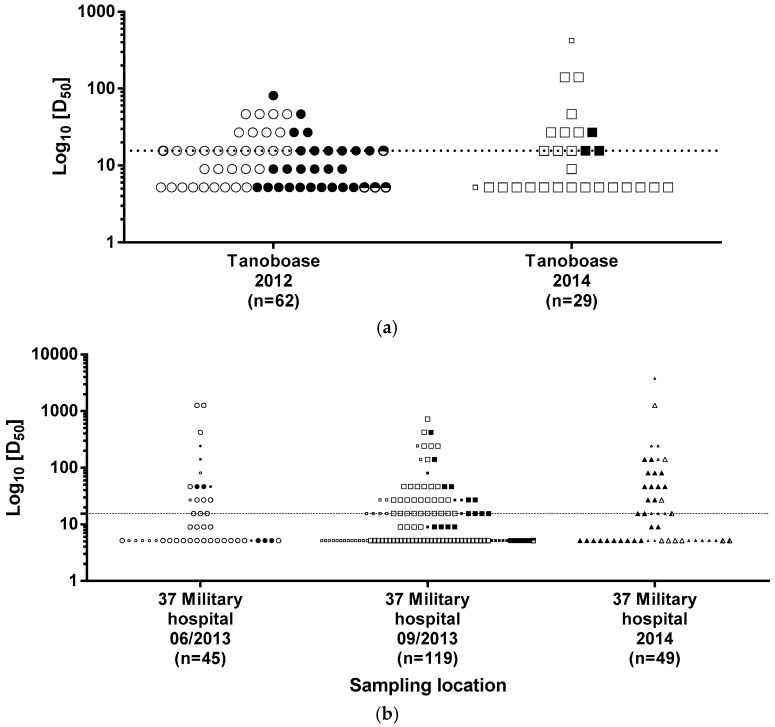
Serological profiles of straw-colored fruit bats sampled at (**a**) Tanoboase in the Brong-Ahafo region and (**b**) at 37-Military Hospital, Greater Accra. Open symbols represent male bats, closed symbols represent female bats; half-filled symbols represent bats of undetermined sex. Smaller symbols represent juvenile or sub-adult bats. The D_50_ value considered the cut-off for positivity is indicated by a dashed line.

Samples taken and data regarding age, sex and serology are detailed in Table 1. A total of 304 bat sera were collected and tested for LBV neutralization antibodies during the sampling periods described. Of the 304 sera tested, 44.7% (*n* = 136) were considered seropositive (Table 1; Figure 2). This comprised 213 from the 37-Military Hospital in Accra and 91 from the Tanoboase roost site, with 236 adults, 36 sub-adults, 22 juveniles and 10 bats of undetermined age (morphometric data not recorded); 210 males, 84 females and 10 of undetermined sex being tested across the two sites (Table 1). Seroprevalences were high during the three years of sampling (2012—46.8%, *n* = 62; 2013—42.7%, *n* = 164; 2014—47.4% *n* = 78). Of those bats that tested serologically positive, 45.2% were known to be male (*n* = 95/210) while 61.9% were identified as female (*n* = 52/84). Results from samples collected from bats of undetermined sex or age were not included in the statistical analyses. There was no significant difference in the different age groups that tested positive (*p*-value = 0.78) (Table 2). Seroprevalence in adult bats (0.46, *n* = 236) was slightly higher than in the sub-adult and juvenile bats combined (0.43, *n* = 58).

In June 2013, two nursing *E. helvum* dams (ID: 900 and ID: 905) were caught with their pups (ID: 901 and 904, respectively) at the 37-Military Hospital roost site. All four animals were apparently healthy with a good body condition and no outward signs of disease. Following serological assessment, the two mothers were both seronegative with a D_50_ of 5.20. In contrast, both pups were seropositive with detectable anti-LBV neutralizing antibody D_50_ of 46.77 and 81.00 for animals 901 and 904, respectively.

Overall, there was no statistically significant difference in seroprevalence between the two sites (Table 1). At 37-Military Hospital, 44.1% (*n* = 94/213) were seropositive (Figure 2a) compared to 46.1% (*n* = 42/91) of bats at Tanoboase (Figure 2b) (chi-square test = 0.04; odds ratio (OR) = 1.085; relative risk (RR) = 1.046; *p* = 0.84).

To assess potential seasonal variation, an evaluation of seropositivity between the two sites was made for each sampling time. From the total sample set obtained for the two sites, there was no statistical significance in the seasonal difference in seroprevalence between the dry season (December–March) and the rainy season (April–November) (*p* = 0.83) (Table 3).

### 4.2. Necropsy Findings in E. helvum Bats

Forty-six bats were examined post-mortem, of which 38 bats had been collected from the 37-Military Hospital roost and eight bats collected from Tanoboase. Thirty-three bats were found dead, while 13 bats were found moribund and were euthanized, following clinical assessment, on welfare grounds (Table 4). Most moribund bats (*n* = 11) had traumatic injuries of unknown origin. All brain samples were negative for lyssavirus antigen and RNA using FAT and the RT-PCRs, respectively.

## 5. Discussion

A total of 304 bat sera was collected and tested for LBV neutralizing antibodies from 2012 to 2014. Of the 304 sera tested, 136 tested positive (seroprevalence: 44.7%) (Table 1; Figure 2). Seroprevalences were high during each of the three sampling years (2012—46.8%, *n* = 62; 2013—42.7%, *n* = 164; 2014—47.4% *n* = 78). These results, taken together with similar LBV serology results reported previously [2], demonstrate the long-term endemic circulation of LBV in the Ghanaian *E. helvum* population. Previous studies have reported serological evidence of exposure to LBV in different *E. helvum* populations, including in Nigeria (14–44% seropositivity; *n* = 140), Ghana (37% seropositivity; *n* = 66), and Kenya (40–67% seropositivity; *n* = 102) [15,17,18]. This is the first time a previously-described seropositive population has been re-tested several years later and has been shown to maintain a similar level of seroprevalence. The mechanism of maintenance for lyssaviruses in bat species with very high roost numbers and population densities is largely undefined, although several variables including population size, contact rates, species susceptibility, immunological competence, and roost structure may all contribute to virus maintenance via an as-yet-undefined mechanism [8,20].

The size of populations involved within the roosts studied and the movement between defined roosts and other roosting sites that remain undefined during migratory periods may influence virus exposure and hence seropositivity. There was no difference in seroprevalence between the two field sites when assessing data comparing sex and age of sampled bats. However, this species is migratory and individuals may travel long distances to feed [21] perhaps enabling interaction between *E. helvum* populations and possibly also those of other bat species.

Of the 294 sera from wild-caught bats tested of known age class, 134 (45.5%) were serologically positive for LBV neutralizing antibodies. There was no significant difference in the seroprevalence detected between the different age groups tested. The slightly higher proportion of seropositive adult bats (0.46) over juvenile bats (0.43) are similar to levels reported in studies addressing serological positivity to RABV in hoary bats (*Lasiurus cinereus*) [22,23]. The observation of two serologically-positive pups from dams that were serologically negative is unexplained. Maternal antibody transfer is unlikely in the absence of detectable circulating antibodies in the dams. Whilst both dams were seronegative, it is possible that their neutralizing antibodies were below the detection limits of the test applied. The pups might also have acquired the antibodies from other seropositive mothers (through allosuckling) or survived infection and thus acquired immunity.

The effect of migration and coloniality on infection dynamics is not known. Reduced migration and population connectivity has been suggested as a mechanism that could increase the amplitude of seasonal outbreaks and increase the probability for spillover of pathogens to other species, such as human beings [24]. The absence of any seasonal difference in LBV seroprevalence was surprising as increased interactions between bats, and hence an increase in pathogen transmission, might be expected during the dry season in Ghana, when there are increased numbers and an increased population density at the roosting sites. It is possible, though, that the bats have similar aggregation and colonial roosting behaviors during migration as observed during the dry season in Ghana. If so, this would account for the lack of variation in seroprevalence observed in this study.

The current understanding of lyssavirus serological positivity within bat populations is poor. Persistence of neutralizing antibodies to lyssaviruses for longer than 12 months has been described previously [25,26]. The long duration of antibodies detected in bats may reflect repeated exposure and may explain the elimination of notable seasonal serological fluctuation in wild-caught bats. Such bats will also influence the epidemiology of an outbreak in the population. From such a small sample set, however, strong conclusions cannot be made and there is a need for a more extensive study to assess serodynamics in bat populations.

The distribution of LBV lineages across Africa is diverse (reviewed in [8]). In Ghana, only serological evidence of LBV has been previously reported, and then only using neutralization assays with lineage B LBV as the test virus [2,15]. The data generated here were also based on using LBV B as the test virus for neutralization. Future studies might benefit from assessment against all four lineages of LBV as well as against other lyssavirus for the potential detection of cross-neutralizing antibodies. Indeed, interpreting serological responses to lyssaviruses is problematic due to the potential for exposure, and serological responses to, undiscovered virus(es). It is possible that further related lyssaviruses exist, confusing the interpretation of these serological outputs. Nevertheless, numerous bats demonstrated significant neutralizing antibody titres against the test virus. This must indicate prior exposure to an LBV or to an antigenically-related lyssavirus. 

Where higher titres are seen, it may indicate repeated exposure to antigenically-similar viruses that drive an anamnestic response. A multiple exposure infection study with rabies virus in bats [26] demonstrated that repeated infection may confer significant immunological memory and reduced susceptibility to RABV infection. It is, therefore, unknown if high seroprevalence is driven by exposure to sub-lethal doses of LBV via routes other than biting, such as from aerosol, grooming or food sharing. The significance of lyssavirus seropositivity in bat populations currently remains undefined.

In an attempt to detect live virus in bats at each study site, diseased and dead bats were assessed for the presence of virus antigen and nucleic acid in brain material. Clinical signs observed in moribund bats included paralyses, ataxia, weakness, vocalization and self-urination. The inability to detect LBV antigen or RNA in the brain samples of moribund or dead bats supports previous findings [27] that the infection prevalence of lyssavirus in gregarious colonial bat species is usually less than 1% of the population. The assays used for molecular detection of viral RNA in the present study are pan-lyssavirus in design and have been assessed to ensure that they are able to detect all lyssaviruses described to date. Many of the necropsied bats were found with evidence of trauma and might have been injured via intra-specific aggression, predators or human hunters; *E. helvum* is widely hunted in Ghana [28]. Many bats, however, had no evidence indicating possible cause of illness or death. A much larger sample size of dead or moribund bats may improve the chances of detecting infection with LBV or a related lyssavirus. A similar study in Ghana with a much higher sample size (*n* = 567) succeeded in isolating LBV in one *E. helvum* bat [29].

## 6. Conclusions

A high prevalence of antibodies against Lagos bat virus (LBV) in *E. helvum* and other bat species was previously demonstrated in Ghana [15,30]. Further follow-up studies confirmed the endemicity of LBV in *E. helvum* in Ghana [2]. The current study reports continued high antibody prevalence levels that support earlier findings. The high seroprevalence of LBV reported previously and in this study in apparently healthy *E. helvum* colonies provides evidence of long-term maintenance and circulation of LBV in this bat population. Demonstrating the maintenance of antibody titres within bat populations is important as it indicates that individual bats are being re-exposed to virus, which in turn suggests that bats are shedding virus and are exposing conspecifics. Whilst this perhaps is not surprising, demonstrating what appears to be a stable level of seroprevalence in a population is important. The mechanisms of transmission of bat lyssaviruses remain ill-defined but minimally, the data presented here reiterate that virus continues to circulate within the bat population, which is important in our understanding of the relationship between bats and lyssaviruses. 

Ongoing studies have indicated the occurrence of close interactions between chiropteran, domestic animal, and human populations. Current methods used to confirm rabies virus infection in people and terrestrial carnivores are unable to differentiate between different lyssavirus species. As human consumption of bats is practiced in many parts of Ghana [30], the potential for human infection with LBV exists and the invariably fatal outcome of lyssavirus infection poses questions as to the risk from bat populations. Importantly, existing rabies vaccines do not fully protect against some African non-rabies lyssaviruses including LBV, Mokola virus, Shimoni bat lyssavirus, Ikoma lyssavirus and West Caucasian bat virus [31,32,33,34,35]; therefore an understanding of the potential risk of transmission of these viruses to human populations is required. The results presented here are of relevance to public health, although further information is required on LBV infection dynamics within bat populations, potential spillover mechanisms, and bat population dynamics, to further define the risk of human infection.

## Figures and Tables

**Table 1:** Sample data collected.

		37-Military Hospital			Tanoboase		Totals
	Sampling Date	10-06-2013	04-09-2013	14-07-2014	17-12-2012	01-07-2014	
**Age**	Adult	34	87	32	57	26	236
	Juvenile	11	6	3	0	2	22
	Sub-adult	0	25	11	0	0	36
	Not determined	0	1	3	5	1	10
**Sex**	Male	34	88	30	33	25	210
	Female	11	30	16	24	3	84
	Unsexed		1	3	5	1	10
**Serostatus**	Positive	17	53	24	29	13	136
	Negative	28	66	25	33	16	168

**Table 2:** Seroprevalence in wild-caught bats by age.

	Total Sampled	Total Positive (% Positive)
**Adult**	136	109 (80.15%)
**Juvenile**	58	25 (43.10%)
**Total**	294	134 (45.57%)

**Table 3:** Proportion of seroprevalence in wild-caught bats by season.

	Positive (+ve)	Negative (−ve)	Total Tested
**Dry Season**	29 (21%)	33 (20%)	62 (20%)
**Rainy Season**	107 (79%)	135 (80%)	242 (80%)
**Totals**	136 (45%)	168 (55%)	304

**Table 4:** Necropsy findings in dead/moribund *E. helvum* bats. All bats found sick were terminated humanely and blood taken as described.

Bat ID	Location	Date Found	Condition	Comments
A1	37-Hospital	20/01/2013	Found dead	N.A.D. *
A2	37-Hospital	20/02/2013	Found dead	N.A.D.
A3	37-Hospital	20/02/2013	Found dead	N.A.D.
A4	37-Hospital	20/02/2013	Found dead	N.A.D.
A6	37-Hospital	28/10/2013	Found dead	Very enlarged spleen
A5	Accra Ridge	29/09/2013	Found dead	Found dead
A12	37-Hospital	27/11/2013	Found sick	Paralysed
A13	37-Hospital	27/11/2013	Found dead	Fractured right arm
A7	37-Hospital	07/12/2013	Found sick	Paralysis
A8	37-Hospital	07/12/2013	Found sick	Paralysis
A9	37-Hospital	07/12/2013	Found dead	Abscess on neck
A10	37-Hospital	10/12/2013	Found dead	N.A.D.
A11	37-Hospital	08/12/2013	Found dead	Fractured forearm
A14	37-Hospital	11/12/2013	Found dead	Fractured forearm
A15	37-Hospital	11/12/2013	Found dead	Severe intestinal, abdominal haemorrhages
A16	37-Hospital	11/12/2013	Found dead	Fractured head
A17	37-Hospital	13/12/2013	Found sick	Paralysis
A18	37-Hospital	13/12/2013	Found dead	N.A.D.
A19	37-Hospital	13/12/2013	Found dead	Punctured wing
A22	37-Hospital	22/12/2013	Found sick	Paralysis, severe haemorrhages on head
A23	37-Hospital	22/12/2013	Found dead	N.A.D.
A24	37-Hospital	22/12/2013	Found dead	N.A.D.
A20	37-Hospital	03/01/2014	Found dead	N.A.D.
A21	37-Hospital	03/01/2014	Found dead	Emaciated carcass
A25	37-Hospital	22/01/2014	Found dead	Fractured forearm
A26	37-Hospital	22/01/2014	Found dead	Decomposed carcass
A27	37-Hospital	22/01/2014	Found dead	N.A.D.
A28	37-Hospital	29/01/2014	Found dead	N.A.D.
A29	37-Hospital	05/02/2014	Found dead	N.A.D.
A30	37-Hospital	12/03/2014	Found dead	N.A.D.
A31	37-Hospital	12/03/2014	Found dead	N.A.D.
A32	37-Hospital	12/03/2014	Found dead	N.A.D.
A33	37-Hospital	12/03/2014	Found dead	N.A.D.
A34	37-Hospital	12/03/2014	Found dead	N.A.D.
A35	37-Hospital	12/03/2014	Found dead	N.A.D.
A36	37-Hospital	06/04/2014	Found sick	Paralysis, emaciated
A37	37-Hospital	06/04/2014	Found sick	Paralysis
A38	Achimota	09/07/2014	Found dead	N.A.D.
T1	Tanoboase	22/01/2013	Found dead	N.A.D.
T2	Tanoboase	22/01/2013	Found sick	Fractured forearm
T3	Tanoboase	22/01/2013	Found sick	Fractured forearm
T4	Tanoboase	23/01/2013	Found dead	Multiple body injury
T5	Tanoboase	23/01/2013	Found sick	Fractured forearm
T6	Tanoboase	07/03/2013	Found sick	Weak
T7	Tanoboase	07/03/2013	Found sick	Multiple body injury
T8	Tanoboase	07/03/2013	Found sick	Weak

* N.A.D.—no abnormality detected
